# Supplementary material for: Modelling disease mitigation at mass gatherings: A case study of COVID-19 at the 2022 FIFA World Cup
Source: PLoS Comput Biol. 2024 Jan 18;20(1):e1011018. doi: 10.1371/journal.pcbi.1011018 (PMC10796029; doi:10.1371/journal.pcbi.1011018)
Supplement: Attachment — Submitted filename: Response to reveiwers comments.pdf [file pcbi.1011018.s005.pdf]

Martin Grunnill
Laboratory of Industrial and Applied Mathematics,
York University, 8 The Chimneystack Rd, Toronto,
Ontario, M3J 3K1
✉ *grunnill@yorku.ca*

To
Editorial Team,
PLOS Computational Biology

September 16, 2023

Revision of manuscript titled “Modelling Disease Mitigation at Mass Gatherings: A Case Study of COVID-19 at the 2022 FIFA World Cup".

Dear Dr Eric HY Lau, Dr. Virginia Pitzer and the PLOS Computational Biology editorial team

I and my fellow co-authors would like to thank the reviewers and editorial team for their feedback that has led to improvements seen in this revised manuscript. Particular thanks goes to Reviewer 1 for their in depth feedback. Below we quote or summarise the salient points made in the editorial team's and reviewer's feedback, followed by outlining the changes we made in reply to and our response to these points.

Yours Dr. Martin Grunnill

Response to Editorial Teams Comments

- “reconsider if fixing the VE waning effect in the sensitivity analysis would lead to increasing protection over time."
- “R0 was assumed to be 2 to 7, referring to a paper published in 2021. However, the Omicron variant was predominantly circulating in the study period, with an R0 of around 10. The assumed range was too low and should be reassessed."

The sampling ranges for these parameters have been altered accordingly and simulations have been redone with new parameter values, see:

- Table 3.
- Figures 4-5.
- S1 Results, S1 Table and S2 Table.
- Abstract.
- Lines 266-277, 309-326, 357-361 and 364-367 of the tracked changes manuscript.

- “Could the development of respiratory symptoms trigger testing in practice? Would that affect the study findings?"

Such a testing regime would be worth comparing to the testing regimes studied. However, in order to study such a policy our model would have to become more complex. A section has been added to the discussion addressing this, see:

- Lines 433-449 of tracked changes version of the manuscript.

Response to Reviewer 1's Major Comments

1: The author's treated each potential world cup match as a 7 day independent Mass Gathering Event (MGE) instead of choosing to model the entire world cup. This was stated in the methods

but needs to be further emphersised.

We have done this, see:

- The line "(modelling each match as independent 7 day MGEs)" has been added to the abstract and Author summary.
- Lines 40-41 in the introduction of tracked changes version of the manuscript.

2: Reasons for excluding matches with the host team from modelling should be stated in discussion.

A new section of the discussion outlines this limitation of our study and the reasons behind this choice, see:

- Lines 422-432 of tracked changes version of the manuscript.

3: “The eight stadiums hosting matches have estimated capacities ranging from 40,000 to 80,000 (45). We assume therefore that the population attending simulated fixtures ranges from 4,000 to 80,000. Were there World Cup matches where attendance was as low as 10% of the stadium capacity? Or was this lower bound simply a means of exploring parameter uncertainty?"

Note that our work on this project began before the World Cup and continued as it was happening. At the time we had difficulty finding projected estimates of stadium occupancy proportion. As such, a range from 0.1 to 1 was used as a means of exploring parameter uncertainty. We now state this, see:

- Lines 157 of tracked changes version of the manuscript.

4: The authors used uniform distributions for parameters sampled using Latin hypercube methods. This is stated briefly in the methodology, but needs to be further emphasized.

This has been done, see:

- The captions of Tables 2, 3, and 5.

5: “In Table 3, the vaccine effectiveness against infection for the *waned* vaccination group is fixed at 0.2230. This value is *higher* than the lower bound for vaccine effectiveness against infection for those *effectively* vaccinated (0.1730). So in about 8% of the simulations, vaccine-acquired immunity would seem to increase over time. Is that correct?"

This is correct and an oversight on our part. Thanks for catching it. Before redoing simulations with an expanded parameter space we corrected this, see:

- Table 3.
- Figures 4-5 of manuscript.
- S1 Results, S1 Table and S2 Table.
- Lines 266-277, 309-326, 357-361 and 364-367 of the tracked changes manuscript.

6: “In Figure 3B, for a small proportion of simulations the testing regimes resulted in greater numbers of infections and hospitalisations than in simulations with no testing regime the percentage difference isn’t always negative. Since the model is deterministic, and the same Latin Hypercube samples were used for each pair of no-testing/with-testing simulations (right?), I’m

struggling to understand how this might occur. Is it possible that the initial state differed between the no-testing and with-testing simulations? Or have I missed an obvious explanation?"

The reviewer's reason is correct. The selection of which stages of infection to place in initial infected population was done via random number generation. Before running new simulations, the underlying code was changed. This initialisation of infected population now accepts a seed number. In the new simulations this seed is generated at the Latin Hypercube sampling stage. As such, positive % relative differences no longer occur, see:

- Lines 193-195 and 207-210 of the tracked changes manuscript.
- Figures 3 and 5.
- S1 Result.

7: "In the discussion, with reference to Figure 6, the authors state: 'The data-set from the (63) [acute cases under hospital treatment] is missing data between 27-10-2021 and 29-6-2022, the data is patchy after 29-6-2022 and no record was made to indicate if a vaccine dose was a second or third booster.' The missing data are not evident in Figure 6, the "Total in Hospital" time-series has no apparent gaps or discontinuities. Were the missing data limited to specific details about each case during this time period, rather than absence of cases?"

The data was very patchy for June through August 2022. In order for a fitted version of our model to produce a reasonable estimate COVID-19 dynamics going into the world cup we would have preferred COVID case and hospitalisation data through the summer of 2022, if not far earlier. As stated in the line 'no record was made to indicate if a vaccine dose was a second or third booster' we also required time series information on full-vaccination or booster vaccination for this fitting. Note that we did update the details of the spotty data in the manuscript, see:

- Line 396 of the tracked changes manuscript.

8: "The authors have made all of their code available in a public GitHub repository, which is great to see. I have explored it a little (e.g., to confirm that all parameter values were being sampled from uniform distributions). The code is generally clear and well structured, for which I thank them!"

We appreciated and thank the reviewer for giving this positive feedback on the code repository.

8.1: "I noticed that a number of data files are read using hard-coded paths that are specific to an author's computer (e.g., 'C:/Users/mdgru/[...]') rather than using paths that are relative to the repository (e.g., '../parameters/data_extraction/[...]')."

The code has been reorganised to avoid absolute path references where possible. In saving the data and producing figures new users may need to alter a few lines of code. The readme file was also altered to instruct users of this modification.

8.2: "Also, it would be great if the authors could add a license to the repository (see, e.g., https://choosealicense.com/) so that other people are able to use the code without any potential for copyright issues."

We are looking into this.

9: “Are the authors planning to release a generic implementation of this mass-gathering event framework, in addition to the 2022 World Cup analyses that have been provided in the public GitHub repository?"

We will discuss this matter and consider next steps in this regard.

Response to Reviewer 1’s Minor Comments

1: Change format of “Efficacy of vaccination with regards to hospitalisation for those effectively vaccinated" from “0.837-1" "0.837 to 1".

Done so, see:
- Table 2.

2: In Table 3, I understand the rationale for most of the vaccine rate parameters being set to zero, but shouldn’t the "Rate of waning immunity of vaccination" be non-zero?

Waning of immunity was not considered as the simulation time, 7 days, is short compared to waning of immunity. We have updated the manuscript to mention this, see:
- Line 164 of the tracked changes manuscript.

3: “’a policy requiring visitors to have had a recent COVID-19 vaccination may have prevented the increase in COVID-19 cases and hospitalisations during the world cup.’ Comparing Figure 3A ("Total Infections and Hospitalisation in simulations made with no testing regime") and Figure 5A ("Boxplots of Total Infections and Hospitalisation under ’effective visitor vaccination’") suggests that such a policy would have reduced cases and hospitalisations. It’s less clear to me that this policy would have entirely prevented an increase in cases and hospitalisations during the world cup."

The phrasing we have used was changed, see:
- Abstract.
- Author summary.

Response to Reviewer 2’s Major Comments

1: “I consider this work publishable, however, the choice of which parameters were reported with uncertainty seemed arbitrary so the paper could benefit from a more thorough sensitivity analysis. In particular, changing the test sensitivities would likely give rather different results (noting the RA sensitivity comes from a study which reports that 0.728 is likely an overestimate)."

We have considered and compared to a lower value of RA test sensitivity in new simulations made in revising this work. This has resulted in testing regimes using pre-match RA tests being more comparable to their pre-match RT-PCR tests counter parts, see:
- Table 3.
- Figures 4-5.
- S1 Results, S1 Table and S2 Table.
- The abstract.
- Lines 229-233, 248, 266-277, 309-326, 357-361 and 365-367 of the tracked changes manuscript.

Response to Reviewer 2's Minor Comments

1: "Equation (1) does not include waning immunity (i.e. the equations are invalid for v=2)."

The parameter ν_v governs movement from one vaccination group to the next. Therefore, when ν_v is $\nu_{v=effective}$ equation set 1 does have waning of vaccination based immunity (see figure 1B to cross reference information on movement between vaccination groups).

2: "Tables 4 and 7 are difficult to read (alternate shading of rows could help here)."

Thank you for pointing this out. This formatting style has been implemented, please see:

- Table 1-7.

3: "pg. 10 has a typographical error in the bounds for N_A."

Changed please see:

- Table 5.

4: "I'm not sure why Figure 5 has a B and C section, I think they could be combined."

Changed please see:

- Figure 5.
