# Supplementary material for: Modelling disease mitigation at mass gatherings: A case study of COVID-19 at the 2022 FIFA World Cup
Source: PLoS Comput Biol. 2024 Jan 18;20(1):e1011018. doi: 10.1371/journal.pcbi.1011018 (PMC10796029; doi:10.1371/journal.pcbi.1011018)
Supplement: Attachment — Submitted filename: Response to Minor amendments.pdf [file pcbi.1011018.s006.pdf]

Martin Grunnill
Laboratory of Industrial and Applied Mathematics,
York University, 8 The Chimneystack Rd, Toronto,
Ontario, M3J 3K1
✉ grunnill@yorku.ca

To
Editorial Team,
PLOS Computational Biology

November 9, 2023

Revision of manuscript titled "Modelling Disease Mitigation at Mass Gatherings: A Case Study of COVID-19 at the 2022 FIFA World Cup".

Dear Dr Eric HY Lau, Dr. Virginia Pitzer and the PLOS Computational Biology editorial team

I and my fellow co-authors would like to thank the reviewers and editorial team for their feedback that has led to improvements seen in this revised manuscript. Below we quote or summarise the salient points made in the editorial team's and reviewer's feedback, followed by outlining the changes we made in reply to and our response to these points.

Yours Dr. Martin Grunnill

Response to Editorial Team's and Reviewer's Comments

◇ "Would the authors consider applying for open source license on your code to promote sharing and usability?"

An Apache 2.0 open license has been applied, see supplementary methods code on github.

◇ "Figure 5B, the authors may consider using the same axis range or other presentation to facilitate comparison between % vaccinated."

Figure 5B has been changed accordingly to Figure 5B and C, see Figure 5 and its caption.
